# Male incidence rates outpacing females: results from a nationwide analysis on acetabular fractures in Germany

**DOI:** 10.1007/s00402-026-06332-1

**Published:** 2026-05-02

**Authors:** Florian Baumann, Susanne Bärtl, Dominik Szymski, Katja Hierl, Volker Alt, Viola Freigang

**Affiliations:** Department of Trauma Surgery, University Hospital Regensburg, Regensburg, Germany

## Abstract

**Introduction:**

Acetabular fractures are uncommon, but serious injuries. Demographic changes may have a significant impact on planning healthcare structures to improve treatment outcomes. Aim of this nationwide, registry-based retrospective controlled study was to identify incidence trends, demographic characteristics, and care structures of patients with acetabular fractures in Germany.

**Materials and methods:**

We analyzed inpatient data from the Institute for the Hospital Remuneration System (InEK). Based on 52 095 patients with primary diagnosis of an acetabular fracture between 2019 and 2024, we calculated incidence rates for different age-groups and put a spotlight on geriatric acetabular fractures (> 65 years of age).

**Results:**

Incidence rates in patients under 65 years remained stable, whereas patients over 65 years showed a significant age-dependent increase with an exponential rise in men aged 80 + with the highest incidence being 122.4/100 000 inhabitants annually. We recorded high levels of co-morbidity and nursing care dependency for elderly patients after acetabular fracture. Although 43% of patients were treated in hospitals > 500 beds, acetabular fractures were managed across all hospital sizes.

**Conclusions:**

There is a rapidly increasing incidence of geriatric acetabular fractures, predominantly driven by elderly male patients over 80 years. Patients over 65 years are associated with high rates of co-morbidities and nursing care levels.

## Introduction

A fracture of the acetabulum is an uncommon but serious injury. Management of these injuries can be challenging [1]. Demographic changes have led to a shift in the epidemiology of acetabular fractures. The incidence of acetabulum fractures reported in the literature ranges from 1.8 to 40 per 100,000 [2]. Several studies have shown a massive increase in acetabular fractures over the past 20 years [2–5]. The number of fractures in young and middle-aged patients, who typically sustain an acetabular fracture after high-energy trauma, has remained unchanged during the past decades [3, 6]. The rising numbers in geriatric fractures are mainly due to over-ageing of industrial societies. The increase of numbers in this age group is most often a result from low-energy trauma like a ground-level fall representing insufficiency fractures based on reduced bone quality [7, 8]. Registry data show that these patients typically present with an involvement of the anterior column of the acetabulum, like in anterior column fractures or anterior column plus posterior hemi-transverse fractures [9, 10]. Unlike osteoporotic fractures in other body regions that predominantly affect post-menopausal women, geriatric acetabular fractures show a balanced sex distribution [4, 11, 12]. The shift in epidemiology and trauma mechanism has substantial implications for treatment strategies. The change in fracture patterns has led to advancements regarding anterior approaches and anatomic plate systems for fixation of the anterior column [13–15]. Regardless of fracture pattern, advanced age is associated with pre-existing co-morbidities and potentially complicating factors, such as frailty and immobilization. Hence, elderly are more likely to have a poor outcome after acetabular fracture [16, 17]. The German hospital landscape is characterized by a high density of institutions with heterogeneous levels of care and different ownership structures, which contributes to fragmented treatment pathways. As part of an ongoing national health system reform, hospital services are planned to be reorganized into defined levels of care, with the aim of consolidating resources and concentrating complex trauma care in specialized structures [18]. Resource allocation and planning of healthcare services rely on robust, population-based data to forecast future needs in healthcare service requirements. The aim of this nationwide study is to determine the incidence, demographic changes and care structure of patients with acetabular fractures in Germany.

## Methods

For this retrospective, controlled registry study, inpatient case data from the Institute for the Hospital Remuneration System (InEK) for all patients treated as inpatients in Germany from 01/2019 to 12/2024 were evaluated. Using the InEK data browser, all inpatient treatment cases in Germany were searched according to diagnosis-related case groups (G-DRG system). All patients with the primary diagnosis “acetabular fracture (ICD-10: S32.4)” were included. Patients were assigned to different age groups and analyzed accordingly (Table 1). We defined the patient group “elderly patients” as 65 years or older. For incidence calculation, the cohort size for each year of age was taken from the population update data of the Federal Statistical Office Destatis (status: 08.01.2025 / 13:16:11). Incidence was calculated as the number of documented cases divided by the at-risk population (sum of population counts for each year of age of the respective patient group at the reference date 31.12. of the respective previous year) and multiplied by 100 000. Thus, the incidence is reported as C/100 000 for an age group.

**Table 1:** Age-related incidence rates between 2019 and 2024

	2019	2020	2021	2022	2023	2024
Incidence rates for all patients						
18–29 years	1931	1614	1553	1729	1811	1843
30–39 years	1982	1799	1729	1818	1803	1866
40–49 years	2436	2455	2671	2751	2443	2562
50–54 years	3898	3726	3576	4145	3598	3951
55–59 years	7032	5907	6117	6441	6094	5894
60–64 years	7754	7879	7992	8508	7530	7944
65–74 years	12,089	11,460	11,505	12,069	11,947	13,263
75–79 years	28,513	27,474	27,105	28,898	30,828	32,743
80 + years	75,764	73,330	73,782	75,790	83,282	87,144
Incidence rates for female patients						
18–29 years	1843	1539	1495	1609	1765	1783
30–39 years	1895	1718	1668	1658	1723	1768
40–49 years	2269	2283	2509	2453	2282	2375
50–54 years	3584	3420	3317	3678	3345	3645
55–59 years	6454	5413	5662	5730	5679	5451
60–64 years	7134	7238	7417	7452	6909	7233
65–74 years	10,837	10,258	10,404	10,171	10,548	11,621
75–79 years	24,407	23,481	23,402	23,300	26,039	27,445
80 + years	56,640	54,736	55,635	54,771	63,050	65,470
Incidence rates for male patients						
18–29 years	2012	1685	1607	1838	1852	1908
30–39 years	2062	1874	1786	1970	1880	1969
40–49 years	2599	2623	2829	3046	2603	2764
50–54 years	4211	4031	3836	4610	3852	4281
55–59 years	7612	6403	6572	7147	6508	6371
60–64 years	8372	8518	8566	9591	8168	8722
65–74 years	13,404	12,724	12,663	14,178	13,507	15,177
75–79 years	33,268	32,098	31,393	35,750	36,703	39,455
80 + years	106,248	102,971	102,708	109,264	115,548	122,373

The analysis included all secondary diagnoses to characterize co-morbidities, all procedures, and structural data of the treating hospital, such as number of beds and hospital ownership. Data from individual years were pooled per group to create cohort analyses. The InEK database provides case-based inpatient data without unique patient identifiers. Therefore, inter-hospital transfers, staged procedures, or readmissions could not be linked and may result in multiple entries for the same individual.

### Statistics

Descriptive statistics include frequencies, percentages within a group, and percentages of the total cohort. The chi-square test was used to compare categorical variables between groups. Additionally, we calculated effect size using Cramér’s V. Normal distribution was confirmed beforehand with a Shapiro–Wilk test. A significance level of *p* = 0.05 was assumed. Calculations were performed using SPSS version 30 (IBM, Armonk, NY, USA). For trend analysis of the incidence of 80 + male patients, we used a linear regression model and an exponential model by applying a natural logarithmic transformation to the incidence values and performing linear regression on the log transformed data. We then compared the goodness of fit of both models using the coefficient of determination (R²). Ethical approval or patient consent was not required since the data were provided pooled and anonymized without the possibility of traceability. To avoid traceability of individual data, the InEK excludes single cases with frequencies below four.

## Results

**Fig. 1:**
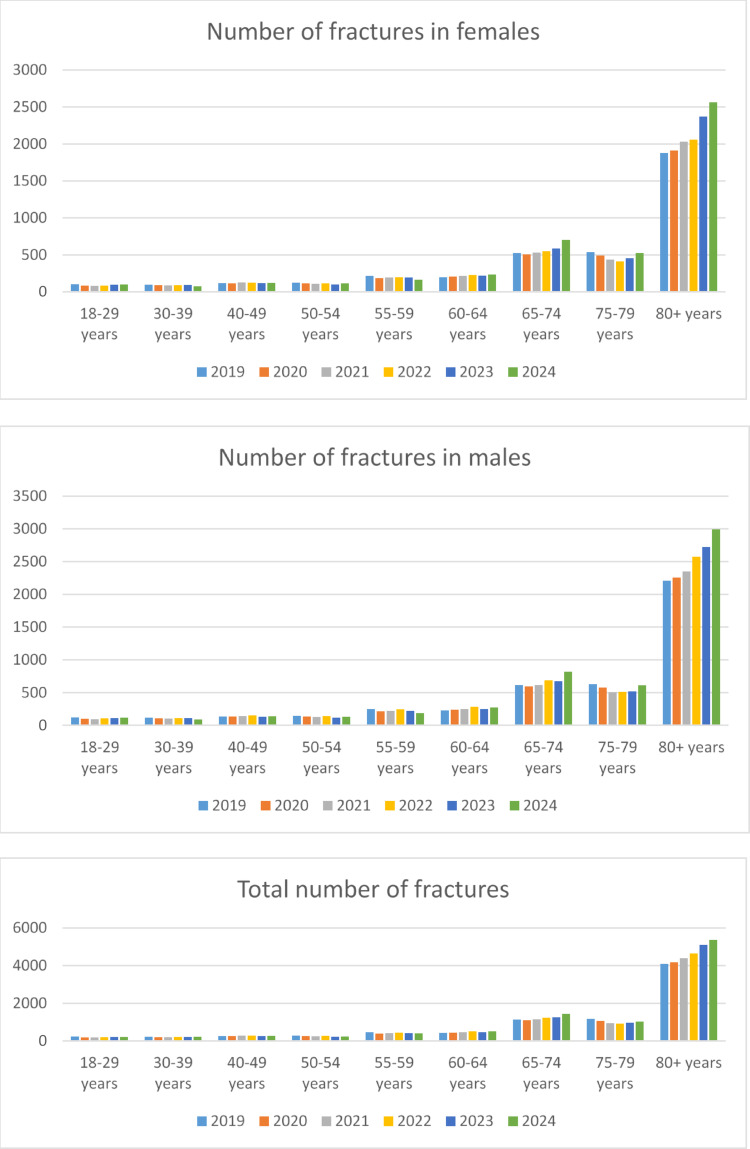
Number of acetabular fractures for each age group

The study analyzes inpatient treatment data of 52 095 patients with an acetabular fracture (S32.4). The study included 23 900 (45.9%) female patients, 28 193 (54.1%) male patients and two patients with undefined sex. Figure 1 shows the numbers of fractures for male and female patients for each age group indicating an exponential rise in patients older than 80 years. The mean incidence of acetabular fractures of all age groups was 15.9/100 000. In 2024, we observed the lowest incidence in the 30–39 year age group at 1.8/100 000, and the highest in patients over 80 years of age with 87.1/100 000 (see Table 1). Men over the age of 80 years had the highest incidence rate with 122.4/100 000 in 2024. The exponential model demonstrated a higher R² than the linear model (0.741 vs. 0.096), indicating that the incidence increased exponentially during the study period. Over the past five years, the incidence in 80 + males increased by 19% and is nearly twice as high as that of women of the same age group (65.5/100 000). Table 2 shows the secondary diagnoses for both groups. 9% of the patients under 65 years had a nursing care level with at least 6 months of impairment compared to 62.5% in the group over 65 years. Table 3 shows the nursing care level at discharge. Regarding diagnostics, younger patients received 2.47 computed tomography scans during the hospital stay, elderly patients over 65 years only 1.53. There was also a difference in the use of contrast-enhanced computed tomography of the pelvis or abdomen, with 5.9% in the elderly and 20.2% in the younger patients (*p* < 0.000). Mean length of stay (LOS) was 12.3 days (± 13.4) for patients under 65 years and 11.7 days (± 9.1) for elderly. Among patients aged 65 years and older, 44.4% were receiving anticoagulative medication (2.6% in under 65 years) (*p* < 0.0000). Regarding operative treatment, 62.6% of patients under 65 years and 41.4% of older patients were treated operatively (*p* < 0.0000). Patients under 65 years received an internal fixation in 6 376/10 992 (58.0%) of cases and only 553/10 992 (5.0%) an arthroplasty of the hip. Over 65 years, 8 231/41 103 (20.0%) of patients received internal fixation (*p* < 0.0000, V = 0.3450), whereas 8 877/41 103 (21.6%) of patients were treated with a replacement of the hip (*p* < 0.0000, V = 0.1756). The transfusion rate was around 20% in both groups (20.1% under 65 and 21.8% over 65). Due to the large numbers, this was statistically significant with *p* = 0.000118, however, an effect size of 0.0169 showed negligible association.

**Table 2:** Secondary diagnoses in patients with an acetabular fracture

DRG-code	Secondary diagnosis	Number of patients (% of age group)	
		Under 65 years	Over 65 years
E87*	Electrolyte imbalance	1721 (15.7%)	12,104 (29.4%)
N39*	Urinary tract infection	590 (5.4%)	9389 (22.8%)
N18*	Kidney failure	203 (1.8%)	8904 (21.6%)
I48*	Atrial fibrillation	204 (1.8%)	10,352 (25.2%)
E11*	Diabetes mellitus	694 (6.3%)	9159 (22.3%)
E03*	Hypothyroidism	623 (5.7%)	5927 (14.4%)
R26*	Immobility	631 (5.7%)	9671 (23.5%)
G20*	Parkinson’s disease	6 (0.1%)	1595 (3.9%)
F32*	Depression	340 (3.1%)	2352 (5.7%)
I50*	Heart failure	149 (1.4%)	8300 (20.2%)
R15*	Incontinence	186 (1.7%)	3938 (9.6%)
I25*	Coronary heart disease	321 (2.9%)	7766 (18.9%)
D50*	Iron deficiency	80 (0.7%)	1206 (2.9%)
R64*	Cachexia	17 (0.2%)	411 (1.0%)
D62*	Anaemia	2016 (18.3%)	7794 (18.9%)
F03	Dementia	5 (0.1%)	5034 (12.2%)
I10.*	Hypertension	2587 (23.5%)	26,384 (64.2%)
Z92*	Anticoagulation	1086 (9.9%)	18,270 (44.4%)
M8*	Osteoporosis	108 (1.0%)	6017 (14.6%)

**Table 3:** Nursing care level at discharge

Nursing care level	Under 65 years	Over 65 years
Grade 1	1.1%	43.3%
Grade 2	3.5%	20.7%
Grade 3	2.0%	19.0%
Grade 4	1.0%	9.0%
Grade 5	0%	1.3%
Grade unknown	1.6%	8.6%
Total	9.4%	62.8%

**Fig. 2:**
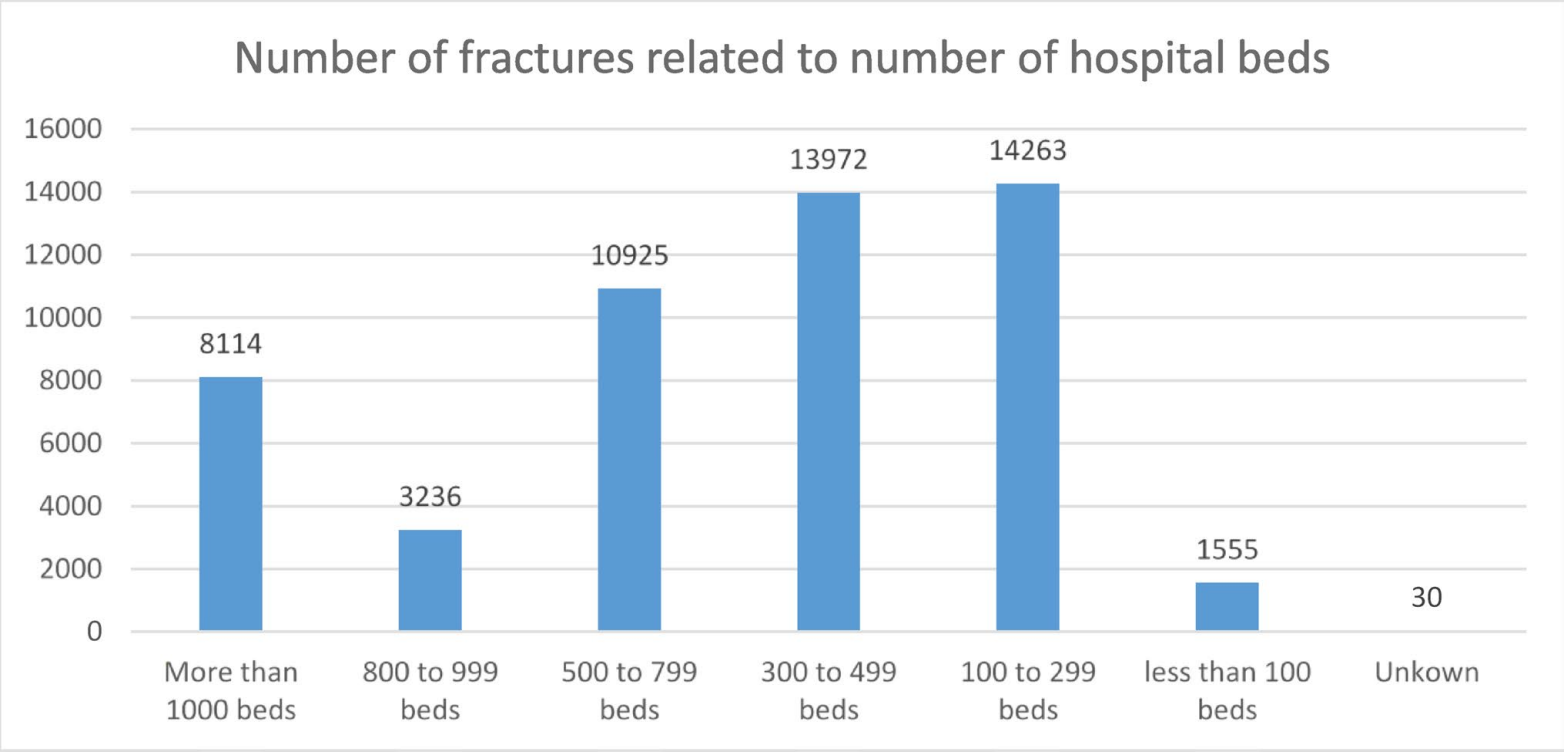
Number of treated acetabular fractures related to the number of hospital beds per institution

Figure 2 shows frequencies of acetabular fractures depending on hospital bed capacity. Hospitals with more than 500 beds treated 42.8% of patients with an acetabular fracture.

## Discussion

This is the first study to report on the actual incidence of acetabular fractures in Germany based on a nationwide healthcare database. The study revealed an unchanged incidence for patients under 65 years of age and a dramatic age-related increase in incidence for patients over 65 years of age between 2019 and 2024. In contrast to geriatric fractures at other body regions, the increase in the incidence of geriatric acetabular fractures is mainly driven by an increase of male geriatric fractures with an incidence of up to 122.4/100 000 in males over 80 years of age. Patients over 65 years are associated with high rates of co-morbidities and nursing care levels.

Several studies across different countries have reported an increase in the incidence of acetabular fractures [2, 3, 6, 11, 12, 19, 20]. Our results are concordant. However, the incidence rate of 122.4/100 000 in men over 80 years is the highest reported in literature and the trend since 2019 shows an exponential growth with 19% increase during the study period. Generally, there is a progressive transition from younger patients sustaining high-energy trauma to geriatric acetabular fractures, typically after low-energy trauma [10, 20–23]. A Swedish study by Lundin et al. found an incidence of 8.7 to 11/100 000 in total. This number increased over the study period from 2001 to 2016 [12]. They observed substantial age- and sex-related differences in incidence rates. Males older than 70 years had an incidence of over 100/100 000. Similar to other studies, the Swedish study found that the majority of acetabular fractures occurred in males (58%). It is notable that the predominance of male patients in this context is not attributable to young males being prone to take risks, since most fractures occurred in the elderly with a mean age of 70 years. A predominance of male geriatric patients for acetabular fractures contrasts with injuries of other body regions, like spinal or pelvic fractures, which typically affect osteoporotic women [7, 24–26]. The reason for the higher proportion of males remains unclear (increase of life expectancy or behavioral). However, it is important to note that there are differences in case registration: The Swedish registry is a national registry tracking each individual patient, whereas our database records all inpatient cases. This may result in multiple entries for the same individual due to inter-hospital transfers, staged procedures, or readmissions.

Lundin et al. recorded surgical intervention only in 15% of patients with acetabular fractures [12]. This contrasts with other registry-based numbers reporting of 62.4%. in 2853 cases of acetabular fractures [10]. In our nationwide analysis, 46.7% of patients underwent a surgical procedure (internal fixation or arthroplasty) of the acetabulum.

Melhem et al. reported on the incidence of acetabular fractures in France between 2006 and 2016 [20]. They recorded an increase in the incidence of 34% to a value of 4.95 per 100 000 people in 2016. The highest incidence was 23.2/100 000 in people over 75 years. The male proportion of all patients was 61% [20].

Studies outside of Europe have shown lower numbers of incidence, however, there is also a trend of increasing numbers of geriatric acetabular fractures and a male predominance [2, 4, 5, 27]. A recent epidemiologic study from Korea reported a lower incidence rate of 3.14 per 100 000 for all age groups and 18/100 000 for people older than 80 years [2]. Although the rates recorded by Park et al. were lower compared to other countries like Sweden and Germany, they also described an increase over the 11-year study period and projected an increase of acetabular fractures of 70% until 2030 [2]. The predominance of male patients was even higher in the Korean population with 62% male. Most patients were treated non-operatively (84%). The characteristics of co-morbidities in the patients within this study match those of our study.

The changes in epidemiological characteristics have affected surgical decision-making on acetabular fracture management. Several authors have outlined the challenges of treatment of geriatric acetabular fractures [9, 15, 28–33]. The fracture pattern of geriatric acetabular fractures is different to fractures in younger patients after high-energy trauma [9, 10]. After high-energy trauma, fractures involving the posterior wall or posterior column, as well as transverse fractures are more common. Acetabular fractures in elderly typically involve the anterior column [9]. Reduced bone quality may lead to comminution zones and reduced anchoring possibilities for fixation of the fracture [34]. Patient-related factors such as co-morbidities and the inability to follow partial weight bearing restrictions are risk factors for peri- and post-operative complications [9, 28, 29]. In younger patients, joint-preserving open reduction and internal fixation is the standard treatment for displaced acetabular fractures [30, 35, 36]. Depending on the treatment goal, non-operative treatment may be a viable option [9, 28]. Although it does not enable the patient for full-weight bearing, percutaneous screw fixation may be an option to increase stability and create a bone stock for later arthroplasty [9, 13]. Other treatment strategies like primary arthroplasty or combined osteosynthesis and arthroplasty (“fix and replace”) may provide sufficient primary stability to allow immediate full weight bearing even in osteoporotic bone [37, 38]. Thus, a combined treatment such as “fix and replace” is associated with a longer OR-time and higher blood loss [29, 30, 37, 39]. Patterson et al. report on a rising proportion of patients by 21.5% with primary arthroplasty for geriatric acetabular fractures (age > 64 years) while ORIF decreased by the same value [29]. In our study the proportion of patients aged over 65 years with primary arthroplasty increased moderately from 21.3% in 2019 to 22.0% in 2024.

Healthcare service in Germany is traditionally characterized by a high density of hospitals and a heterogeneous, fragmented healthcare structure. Ongoing reforms of the German healthcare system aim towards center-based trauma care and adequate resource allocation to improve treatment quality [18, 40]. Although 43% of patients were treated in hospitals with more than 500 beds, our data confirm that acetabular fracture patients are treated in all levels of care institutions. However, the increased incidence of geriatric fractures and associated multi-morbidity burden support the political efforts towards specialized high-volume centers to implement inter-disciplinary, orthogeriatric treatment concepts [32, 41, 42].

Limitations include missing patient-specific factors such as bone quality, fracture morphology or radiological images, and exclusion of outpatient treatment data. Despite this, acetabular fractures are mainly treated as inpatients. For data protection reasons, InEK provided only pooled data, so only aggregate frequencies could be analyzed, and no case-level analyses were performed. For example, it was not possible to determine in which cases patients were treated with a fix-and-replace strategy and which patients underwent only primary arthroplasty. The InEK database provides case-based inpatient data without the possibility for individual patient identification or tracking. Therefore, inter-hospital transfers, staged procedures, or readmissions cannot be tracked and may result in multiple entries for the same individual. Data regarding ICD-diagnoses are only available as pooled prevalence and without attribution of severity. Therefore, calculation of a co-morbidity index like Charlson was not possible. For the same reason, it was not possible to distinguish between isolated procedures like arthroplasty or combined procedures like “fix and replace”. Relevant information for decision-making like fracture morphology or Letournel classification are not included in the InEK dataset limiting applicability of our results. Another limitation is that the care level before the injury was not included in the dataset. Therefore, functional decline attributable to the fracture could not be determined. InEK database provides data only within fixed age-groups. Therefore, we cannot provide age-groups other than those reported in the manuscript and may not discern between the influence of changes in population structure or true age-specific risk. Hospital size as measured by bed count does not reflect an institution’s pelvic trauma expertise or case volume. Finally, these data report only on the in-patient treatment of acetabular fracture patients and do not include longitudinal data or outcome results. Variables with frequencies less than four were excluded to prevent identification. Despite these limitations, the main strength of the study is that the dataset includes all inpatient treatment data for acetabular fractures in Germany.

## Conclusion

The study revealed an unchanged incidence for patients under 65 years of age and a dramatic age-related increase of incidence for patients over 65 years of age. In contrast to geriatric fractures at other body regions, the increase in the incidence of geriatric acetabular fractures is mainly driven by an increase of male geriatric fractures. The incidence of up to 122.4/100 000 in males over 80 years of age is the highest incidence of acetabular fractures ever reported. Elderly acetabular fracture patients are associated with high levels of co-morbidities and nursing care levels. Further studies are needed to investigate the influence of patient-related and radiological factors.

## Data Availability

No datasets were generated or analysed during the current study.
